# Ultrasound-assisted brace casting for adolescent idiopathic scoliosis, IRSSD Best research paper 2014

**DOI:** 10.1186/s13013-015-0037-8

**Published:** 2015-04-11

**Authors:** Edmond H Lou, Amanda CY Chan, Andreas Donauer, Melissa Tilburn, Doug L Hill

**Affiliations:** Department of Surgery, University of Alberta, Edmonton, AB T6G 2B7 Canada; Department of Research and Technology Development, Glenrose Rehabilitation Hospital, Edmonton, AB T5G 0B7 Canada; Department of Prosthetics and Orthotics, Glenrose Rehabilitation Hospital, Edmonton, AB T5G 0B7 Canada

**Keywords:** Adolescent idiopathic scoliosis, 3D ultrasound imaging, Brace treatment, Brace design, Optimum brace pressure

## Abstract

**Background:**

Brace treatment is the most effective non-surgical treatment for AIS. High initial in-brace correction increases successful brace treatment outcomes. The objective of this study was to investigate if real-time ultrasound (US) can aid orthotists in selecting the pad pressure level and location resulting in optimal in-brace correction of the spine.

**Methods:**

Twenty six AIS subjects participated in this pilot study with 17 (2 M, 15 F) in the control group and 9 (2 M, 7 F) in the intervention group. For the control group, the standard method was used to design their braces. In addition to the standard of care, a medical 3D ultrasound (US) system, a custom pressure measurement system and in-house software were used to select pad placement and pressure levels for the intervention group. The orthotist used a custom standing Providence brace design system to apply pressures against the patient’s torso. The applied pad pressures were recorded. A real-time US spinal image was displayed. Cobb angle measurements from the baseline and the assessment scan were performed. The orthotist then decided if an adjustment was needed in terms of altering the pad locations and pressure levels. The procedures may be repeated until the orthotist attained the best simulated in-brace correction configuration to cast the brace.

**Results:**

In the control group, 8 of 17 (47%) subjects needed a total of 16 brace adjustments after initial fabrication requiring a total of 33 in-brace radiographs. For the intervention group, the orthotist tried additional configurations in 7 out of 9 cases (78%). Among these 7 revised cases, 5 showed better stimulated in-brace corrections and were subsequently used to cast the brace. As a result, only 1 subject required a minor adjustment after initial fabrication. The total number of in-brace radiographs in the intervention group was 10.

**Conclusions:**

The use of the 3D ultrasound system provided a radiation-free method to determine the optimum pressure level and location to obtain the best stimulated in-brace correction during brace casting. The average number of radiographs per subject taken prior to final brace implementation with the interventional group was significantly lower than the control group.

## Background

Adolescent idiopathic scoliosis (AIS) is a three-dimensional deformity of the spine associated with vertebral rotation due to an unknown cause. It affects adolescents’ emotional and social wellbeing, and may cause physiological problems in severe cases. If left untreated, AIS may progress and give rise to serious health problems including spine degeneration [1,2], cardiopulmonary compromises [3], negative body image and psychosocial disorders from a grossly deformed torso [4]. Bracing is typically prescribed either based on guidelines set by the Scoliosis Research Society [5] or by the Society on Scoliosis Orthopaedic and Rehabilitation Treatment (SOSORT) [6], in which the Cobb angle is greater than 20° in a child with considerable growth remaining or show at least 5° of Cobb angle increase between consecutive clinic visits. Over the last few years, scientific evidence has shown that brace treatment is effective [7-10], with higher success rates when brace wear time is more than 12.9 hours per day [7]. However, brace wear time is only one factor which affects brace treatment outcomes, others include a) growth or curve based risk, b) the in-brace correction, and c) the wear tightness relative to the prescribed level (quality of brace wear) [11,12].

A spinal brace is a hard plastic shell with pads installed inside the liner to concentrate and direct the corrective pressure to oppose the spinal curvature. There is only a short window of time during adolescent growth for brace treatment to be effective. In standard practice, the in-brace correction is obtained through radiographic measurement typically reviewed within 2 months after the brace has been initiated. If the in-brace correction is not deemed to be satisfactory by the treating orthopedic surgeon, the patient returns to the orthotist for readjustment. This test and adjust is repeated until the results are acceptable or deemed unattainable. This repeated procedure increases cumulative radiation exposure and shortens effective brace usage. Radiation exposure in growing children may increase risk of cancer. Furthermore, in current practice, brace wear tightness and locations of pads are set empirically based on guidelines for the type of the brace, with little scientific support.

Too much force applied at the brace pad site causes discomfort and likely ultimately reduces compliance, but too little force compromises curve correction. In addition, less than optimal pad placement reduces the effectiveness of treatment increasing the likelihood of additional radiographs and ultimately the need for surgery due to brace failure. Finite element (FE) models have been developed to determine optimal orientations and load magnitudes of pressure pads [13,14] but these still have practical limitations [15] with evaluation of the brace correction not available until the in-brace follow-up clinic. Therefore, a real-time non-invasive and non-ionizing method to assess spinal correction during brace construction is essential to overcome the limitations and undesirable consequences associated with the existing methods. Furthermore, there was a study applying ultrasound to determine the optimum location of the major brace pad [16,17], but this approach did not provide real-time feedback nor determine the optimum pad pressure. Their ultrasound data were processed between the time the patient had their brace fitting and returned to receive the modified brace. The objective of this study was to investigate if real-time ultrasound (US) can aid orthotists in determining the pad pressure level and location resulting in optimal in-brace correction of the spine.

## Methods

### Patients

Seventeen retrospective consecutive AIS subjects (2 M, 15 F; age 13.2 ± 1.5 years), who were prescribed new braces including both providence braces and full-time TLSO, were recruited as the control group before the intervention group recruitment. Nine (2 M, 7 F; age 13.3 ± 1.4 years) new AIS subjects who were prescribed either Providence braces or full time TLSO were prospectively recruited into the intervention group. The ethics approval was granted by the University of Alberta Research Ethics Boards and all subjects signed consent forms before participation. The inclusion criteria followed the guidelines set by the non-operational management team of the Scoliosis Research Society [18] a) age 10 years or older when brace is prescribed, b) Risser 0–2, c) primary curve angles 20°-45°, d) no prior treatment, and, e) if female, either premenarchal or less than 1 year postmenarchal.

### Orthotists

Two orthotists were involved in this study; one has 18-years experience and the other one has 7-years experience. The orthotists work together in the same department and cover for each other at the scoliosis clinic. The less experienced orthotist used the same approach as the more experienced orthotist to build spinal braces. In this study, all subjects of the control group were recruited from the more experienced orhtotist’s clinics. For the intervention group, 3 subjects were recruited from the less experienced orthotist and the rest was from the more experienced orthotist.

### Control group protocol

For the control group, the orthotists used traditional plaster cast and molded method with the assistance of the providence brace system to design spinal braces. The orthotist first applied a plaster rigid wrap to the AIS body while the subject is standing. The subject then lays upon the Providence brace system. The orthotist places the pressure pads and applies pressure at each pad based on the location of the curve apex and his/her experience. After the plaster hardens, the patient stands again to remove the hardened cast. The cast was then scanned by a handheld laser scanner to create a positive mold. After subjective modifications for improved fitting and comfort on the positive mold file, a brace was fabricated.

### Intervention group protocol

**Figure 1:**
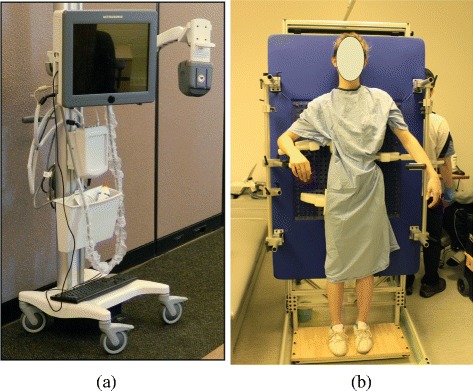
The apparatus for patient data acquisition. **a)** The 3D ultrasound equipment (Left) and **b)** the set up to scan a brace subject.

**Figure 2:**
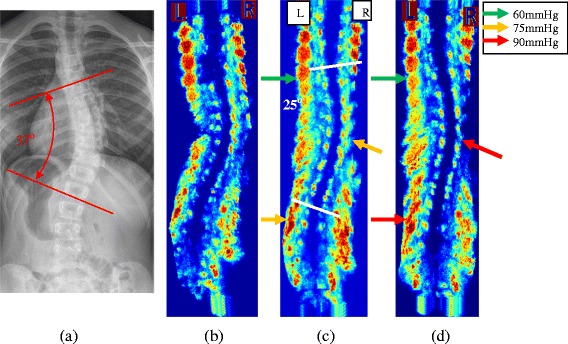
X-ray and US Images of a brace patient. **a)** The standing pre-brace x-ray with Cobb angle 37°, **b)** the baseline US scan, **c)** the 1st trial US scan with Cobb angle 25° and **d)** the 2nd trial US scan with Cobb angle 23. The color arrows indicate the pressure magnitude applied to the body.

A custom Providence brace standing frame, a medical ultrasound (US) system, a custom pressure measurement system, and in-house US measurement software were used to assist brace casting for the intervention group. An opening (14 cm × 50 cm) was cut at the middle of the frame to allow for the ultrasound scanning probe. Figure 1 shows the US equipment and the custom Providence brace design set up with a participant. Each subject put on a gown with their back open and stood against the standing frame. The subject was then scanned by the US system. It took approximately 1.5 minutes to acquire the data and to display the image. The pre-brace x-ray and the standing US spinal image were displayed side-by-side to assist the orthotist to decide pressure pads locations. The orthotist used the custom standing Providence brace design system to secure pressure pads with subjectively determined applied pressure levels against the patient’s torso to simulate in-brace correction. The simulated in-brace US scan was then acquired. A real-time US spinal image was displayed and the Cobb angles were measured using in-house developed software. This process also took less than 2 minutes. The mean absolute difference of the ultrasound measurements compared to the corresponding radiographic measurements was reported in [18] to be 3–4 degrees with good consistency between the imaging modalities. The orthotist then decided if altering pad locations and pressure levels might improve correction. Another US scan was taken if needed. The procedures repeat until the orthotist attained the best simulated in-brace correction configuration. During scanning, the pressure levels at each pressure pad were also recorded wirelessly by a custom pressure measurement system. Figure 2 shows a) the pre-brace standing x-ray, b) the standing baseline US image, c) the first US scan with axilla, thoracic and lumbar pads pressure levels at 60, 75, 75 mmHg, respectively, d) the second US scan with axilla, thoracic and lumbar pads pressure levels at 60, 90, 90 mmHg, respectively. The orthotist then applied a plaster rigid wrap to the participant in a standing position and applied pads pressure levels identical to the best stimulated in-brace correction configuration. After the plaster hardened and was removed, the cast was scanned by a handheld laser scanner to create a positive mold which was used for brace fabrication.

### Ultrasound measurements

To measure the coronal curvature on the ultrasound image, the centers of laminae (COL) method introduced by Chen et al. [19,20] was applied. The centers of laminae at the end vertebra levels were manually selected on the coronal plane of the ultrasound image. Lines were then automatically drawn across the two points, on the left and right, at each of the end vertebrae level (Figure 2c) by our in-house program. A corresponding transverse view image was then displayed to allow fine tuning the position of the centers of laminae. Since the ultrasound data was three-dimensional, any adjustment of the COL on the transverse view would be reflected on the coronal view. The Cobb angle measured on Figure 2c and d using the COL method were 25° and 22°, respectively.

### Pad pressure measurements

To measure the pad pressure during the brace casting clinic, an in-house wireless pressure measurement system which was similar to the pressure control system described in [21] was used. The measurement system consisted of a microcontroller with wireless capability, pressure sensor, an electronic pump and multiple electronic valves. Three air bladders connected to the valves and switching mechanism were used to measure the interface pressures between the body and pads. At the beginning, a small amount of air was pumped into the bladder so that pressure measurements could be obtained. Pressures applied by the orthotists via the pads to the body were transmitted wirelessly and displayed on a laptop computer in real-time.

### First follow-up clinic

Approximately 6 weeks after braces initiation, all participants returned to scoliosis clinics to inspect the effectiveness of the brace. Each subject was requested to have an in-brace radiograph on that day. The treating orthopedic surgeons used the threshold of in-brace Cobb correction for at least 30% to be the minimum acceptable requirement and their experience to further judge if the in-brace correction was optimal. If the surgeon was not satisfied with the in-brace correction, participants would return to the orthotist for adjustments. Additional follow-up clinic visits with radiographs occurred approximately 2 months after adjustments.

## Results

In the 17 control subjects, the major out of brace Cobb angle was 32 ± 7 degrees. Eight of these required brace adjustment (47%). A total of 16 brace adjustments were needed and 33 in-brace radiographs were taken (average 1.9 radiographs per subject). The average in-brace major Cobb angles at the first in-brace follow-up clinic and at the final accepted follow-up clinic were 22 ± 14 degrees and 14 ± 11 degrees, respectively. The average in-brace Cobb corrections were 31 ± 29% at first attempt and 56 ± 27% after final adjustments. For the intervention group, the major out of brace Cobb angle from the radiographs prior to bracing was 29 ± 8 degrees. The orthotist casted the second stimulated configurations in 7 out of 9 cases. The 2 cases which were deemed not to require revised pad placements had in-brace Cobb correction of 65% and 55% on the first trial, respectively. Among the 7 revised cases, 5 showed better stimulated in-brace corrections, 1 had no change and the other one got worse. For the 5 improvement cases, the in-brace Cobb correction from the ultrasound measurements in the first and second trials were 28 ± 13% and 39 ± 12%, respectively. Among the 9 intervention cases, only 1 brace adjustment was deemed necessary at the follow-up clinic visit and 10 radiographs were taken (average 1.1 radiographs per subject). The average final in-brace Cobb angle was 11 ± 13 degrees which was 51 ± 21% in-brace correction. Table 1 shows the comparison of the orthotists time to cast and make the brace adjustment between the control and the intervention groups; on average an extra 0.6 hour/per subject (36 minutes) was needed in the control group.

**Table 1:** **Comparison of the casting and the brace adjustment time per subject**

	**Control group**	**Intervention group**
**Casting time**	17 hours (1 hour per subject)	10.8 hours (1.2 hours per subject)
**Brace adjustment time**	16 hours (1 hour per adjustment)	1 hour
**Total time**	33 hours	11.8 hours
**Average**	1.9 hours per subject	1.3 hours per subject

## Discussion

Brace treatment is the most effective non-surgical method that can stop the progression of AIS. Beside the patients’ compliance, a good brace design which can provide the best in-brace correction is important. In current typical practice, the skill and experience of the orthotist are the major factors to affect the design of the brace. The pressure pads’ levels and locations are subjectively selected by the orthtoist. Without the real-time non-ionizing imaging method in this paper, the pressure pads may not be installed at the best location. Also, too much force applied at the brace pad site causes discomfort and likely reduces future compliance, but too little force compromises curve correction. From this study, 8 out of 17 cases (47%) of the control subjects need brace adjustments and some required multiple adjustments. In the intervention group 5 out of 9 cases (56%) showed the revised pad placements resulted in better correction than the first trial. Without the real-time feedback, these 5 subjects may have required brace adjustments or attained inferior correction with the original pad placements. Requiring brace adjustment increases not only the number of radiographs and the cost of the health care system (orthotists’, surgeons’ and clinics time), but also the burden for the families that they need to travel to both brace adjustment and extra follow-up clinics. Furthermore, the benefits of getting the best designed brace in the shortest time may improve the overall effectiveness of the brace treatment because the patient will be using the brace most effectively sooner, during the most beneficial period of adolescent growth.

The novelties of this study are the use of the ultrasound and pressure measurement systems providing a radiation-free real-time imaging and pressure measurements system which assists orthotists to determine the optimum pressure levels and locations to obtain the best stimulated in-brace correction during brace casting. Since most of the scoliotic patients are adolescent females, the non-ionizing radiation method is especially desirable. Furthermore, the pressure measurement system assists orthotists’ to make a more effective brace by providing them information on how much pressures they should apply and where the pad locations should be.

## Conclusions

The use of the ultrasound system provided a radiation-free method to determine the optimum pressure level and location to obtain the best stimulated in-brace correction during brace casting. The average number of radiograph taken per subject on the interventional group was lower than the control group. Acceptable in-brace correction was attained sooner in the intervention group with less burden on the families and patients.
